# Spanish Transcultural Adaptation and Validity of the Behavioral Inattention Test

**DOI:** 10.1155/2017/1423647

**Published:** 2017-01-11

**Authors:** Ángel Sánchez-Cabeza, Elisabet Huertas-Hoyas, Nuria Máximo-Bocanegra, Rosa María Martínez-Piédrola, Marta Pérez-de-Heredia-Torres, Jorge Alegre-Ayala

**Affiliations:** ^1^Hospital Universitario Fundación Alcorcón, Madrid, Spain; ^2^Department of Physiotherapy, Occupational Therapy, Rehabilitation and Physical Medicine, Universidad Rey Juan Carlos, Madrid, Spain

## Abstract

**Objective:**

To adapt, validate, and translate the Behavioral Inattention Test as an assessment tool for Spanish individuals with unilateral spatial neglect.

**Design:**

A cross-sectional descriptive study.

**Setting:**

University laboratories.

**Participants:**

A sample of 75 Spanish stroke patients and 18 healthy control subjects.

**Interventions:**

Not applicable.

**Main Outcome Measures:**

The Behavioral Inattention Test.

**Results:**

The Spanish version of the Behavioral Inattention Test shows a high degree of reliability both in the complete test (*α* = .90) and in the conventional (*α* = .93) and behavioral subtests (*α* = .75). The concurrent validity between the total conventional and behavioral scores was high (*r* = −.80; *p* < 0.001). Significant differences were found between patients with and without unilateral spatial neglect (*p* < 0.001). In the comparison between right and left damaged sides, differences were found in all items, except for article reading (*p* = 0.156) and card sorting (*p* = 0.117).

**Conclusions:**

This measure is a useful tool for evaluating unilateral spatial neglect as it provides information on everyday problems. The BIT discriminates between stroke patients with and without unilateral spatial neglect. This measure constitutes a reliable tool for the diagnosis, planning, performance, and design of specific treatment programs intended to improve the functionality and quality of life of people with unilateral spatial neglect.

## 1. Introduction

Unilateral spatial neglect (USN) is a cognitive spatial disorder that can affect perception and the mental representation of spatial information, as well as the ability to plan and perform motor actions. This is, in addition to, the inability to respond, orientate, or react to stimulation received on the opposite side of the damaged hemisphere [1]. This disorder is experienced by 23–43% of stroke survivors and is most common in right hemisphere strokes (43% right hemisphere; 20% left hemisphere) [2, 3]. Interestingly, although some patients with neglect recover completely within the acute poststroke period, in other cases, neglect persists [4, 5]. This impairment is one of the most disruptive factors affecting the recovery of functional independence in stroke patients [1, 6].

*Functional Implications of Unilateral Spatial Neglect.* Patients with neglect suffer from problems which affect their daily activities, including difficulties with personal hygiene, dressing, eating, swallowing, movement and mobility, understanding, and reading [7]. The behavioral signs most commonly associated with neglect include, amongst others, an inability to communicate with people on the neglected side, attending to only one side of the body as far as hygiene and personal cleanliness are concerned (such as only shaving one side of the face or only washing one side of the body), and bumping into people and objects on the affected side. Patients frequently get lost and are unable to find their way. Also, they ignore food on one side of their plate and cannot copy or draw an object without omitting or distorting a part of it. Furthermore, many patients fail to read letters or words on one side of the page.

*Evaluation of Unilateral Spatial Neglect.* The neuropsychological evaluation of neglect is complex, as patients often have other associated cognitive disorders that hamper examination and diagnosis [8]. Several types of tests have been used to assess neglect, mainly based on visual scanning activities [9, 10], as well as line bisection tests [11], cancellation tests [12], drawing tests [13], reading tests [14], and bilateral simultaneous stimulation [15]. None of these tests evaluate aspects relating to everyday life.

*The Behavioral Inattention Test.* The original Behavioral Inattention Test (BIT) was designed by Wilson, Cockhurn, and Halligan in 1987, based on proven graphic visual scanning tests, complemented by a series of tests based on everyday tasks, in order to provide a more natural and ecological testing environment. The BIT is divided into two sections, the first of which is conventional and comprises six tests: line bisection, star cancellation, copying figures and shapes, line cancellation, representational drawing, and letter cancellation. The second behavioral section comprises nine tests selected by psychologists and occupational therapists and based on specific difficulties faced by patients with visual neglect in everyday life situations, such as picture scanning, phone dialing, menu reading, article reading, telling and setting the time, coin sorting, address and sentence copying, map navigation, and card sorting. The BIT therefore combines a series of parameters capable of providing accurate data to reflect the functional incapacity of a person suffering from neglect [16].

The information provided by the BIT is important for the recovery of stroke patients, whose difficulties extend hospital stays and impact the recovery of motor skills [17]. It is also fundamental for proper planning of occupational therapy treatment, as it deals with the functional consequences caused by cognitive deficits [18].

When this instrument is used to evaluate non-English speaking patients (the language in which the BIT was designed), transcultural adaptation is required to ensure semantic, linguistic, and cultural equivalence, particularly regarding items that have a clear and specific cultural background (coins, cards, food, etc.). This article describes the process of academic translation and transcultural adaptation of the BIT to Spanish and analyzes the validity and reliability of the BIT in Spanish stroke patients with, and without, USN. Our work hypothesis is that this adaptation of the scale will enable clinicians to detect neglect in Spanish stroke patients. Also, we hypothesize that the scores in conventional and behavioral test in patients with USN will be significantly worse than stroke patients without neglect.

## 2. Material and Methods

### 2.1. Study Design

This is a cross-sectional descriptive study designed to adapt, translate, and validate a Spanish version of the BIT.

### 2.2. Sample

The study was carried out in the Autonomous Community of Madrid, Spain, and included a sample of 93 subjects, of whom 75 were patients and 18 were healthy controls. Patients were recruited from various multidisciplinary neurological rehabilitation centers: POLIBEA, Centro Estatal de Atención al Daño Cerebral, and the Amma Home for the Elderly (Alcorcón). The inclusion criteria for the study group consisted of a diagnosed stroke with minimal evolution of 6 months and proven unilateral damage and being over the age of 18 years. The doctors and neuropsychologists at the centers were in charge of selecting the sample. The exclusion criteria were the presence of other central nervous system disorders (tumors, anoxia, etc.), cardiorespiratory or neurodegenerative conditions, severe brain damage, and severe awareness deficits. Healthy subjects were recruited from the researcher's social environment and the relatives of the stroke patients. The inclusion criteria included subjects over the age of 18 years and exempt from any condition that may limit their functional capacity. Participants with USN were distinguished by a neuropsychologist and an occupational therapist who participated in the intervention according to daily performance after stroke.

### 2.3. Adaptation Process

Permission was firstly obtained from the authors of the test for the translation and validation of the same to the Spanish population. We then obtained authorization from the Rey Juan Carlos University Ethics Committee.

The translation process was carried out according to the study published by Beaton et al. [19] as follows:

- Translation into Spanish: the test was translated by two native-speaking Spanish translators, resulting in translations T_1_ and T_2_.
- Analysis: both translations were analyzed to reach a consensus on a single translation (T_12_).
- Back translation to English: native-speaking English translators, who were unaware of the process carried out, then translated T_12_ into two new English versions (RT_1_ and RT_2_).
- Revision by an expert committee: the committee was comprised of four occupational therapists, two translators, and two researchers familiar with the tool. After analyzing all the versions (T_1_, T_2_, T_12_, RT_1_, and RT_2_), a prefinal Spanish version was chosen.
- Pilot testing of the prefinal version: in this phase, the prefinal version was used on a sample of patients (30–40), who were subsequently interviewed regarding any difficulties they had in understanding the meaning of the questions and the responses. We also analyzed incidents of noncompleted and repeated replies (i.e., when all patients provide the same response to a specific question).
- Use of the tool: the present pilot test was designed and the tool was administered to 93 people, including 75 patients and 18 healthy control subjects.
- Conclusions: we checked for errors and typing mistakes which derived in the final version of the test. This version was then sent to the authors of the original test.

**Figure 1:**
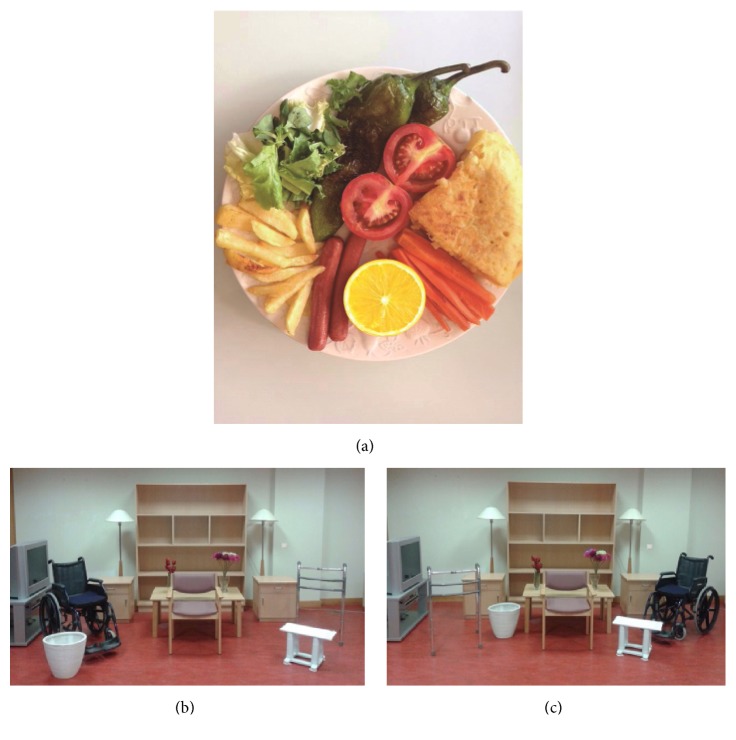
Picture scanning test. Adaptation to the Spanish population (authorized by Pearson Assessment).

With regard to the changes made to the test for the Spanish population, firstly, all the tests requiring words in Spanish were translated, as follows: for the conventional subtests: star cancellation (*cancelación de estrellas*); for the behavioral subtests: menu reading (*lectura de un menú*), article reading (*lectura de un artículo*), coin sorting (*clasificación de monedas*), and address and sentence copying (*copia de una dirección y una frase*). Adaptations to the behavioral picture scanning test were made for the Spanish population: specifically, we adapted picture scanning test. In Figure 1(a), the most common Spanish foods were added, such as green peppers, Spanish omelette, French fries, and lemon (Figure 1). In Figures 1(b) and 1(c), an attempt was made to use the same original furniture, although other simple elements are added (Figure 1). Changes were made in the coin sorting (*clasificación de monedas*) test and the euro was featured, which is the currency used in Spain. Finally, the card sorting (*clasificación de cartas*) test was changed and a Spanish deck of cards was used instead of a poker deck.

### 2.4. Statistical Analysis

Given that the size of the compared groups did not comply with consistency criteria, the Mann–Whitney nonparametric test was used to study the differences between independent samples.

Firstly, we obtained descriptive data, which shows the frequency of the categorical variable, the median, and the interquartile range (q1–q3) of the other continuous variables, as well as the estimated reliability of the assessment tool using the Cronbach *α* test, which indicated adequate reliability (Table 4). Secondly, we analyzed the dependent variables to determine the differences between groups in a cross-sectional manner for all the subjects included in the study. Results with *p* values < 0.05 were considered statistically significant.

All computations were conducted using IBM SPSS Statistics 19 for Windows (IBM Corporation, Armonk, NY, US).

## 3. Results

Table 1 shows the sociodemographic data of the entire sample, divided into the following subgroups: right stroke, left stroke, and healthy control subjects.  Table 2 presents the sociodemographic data of participating patients with and without unilateral spatial neglect.

Table 3 displays the means and standard deviations of the data obtained from the healthy control subjects, as well as the cut-off point. The scores obtained by the control group were used to establish the limits of the normal distribution and determine the score that would serve as a cut-off point for the individual test components.

With regard to the reliability of the scale, the complete test was found to be highly reliable (*α* = .90) as were the conventional (*α* = .93) and behavioral subtests (*α* = .75) (Table 4). The concurrent validity of the test as a measure of spatial neglect was determined by the relationship between the conventional and behavioral total scores obtained by the 75 stroke patients, with a correlation of −0.80 (*p* < 0.001).

Finally, we found statistically significant differences between patients with and without unilateral spatial neglect in all the items on the BIT (*p* < 0.001) (Table 5). In the comparison between right and left brain injury, we found differences in all the items except article reading (*p* = 0.156) and card sorting (*p* = 0.117) (Table 6).

## 4. Discussion

The objective of this study was to create a Spanish adaptation and translation of the BIT (BIT-E) for use by patients with USN and to analyze the validity and reliability of the same in Spanish stroke patients with and without USN. Moreover, we hypothesized that the conventional and behavioral scores of USN patients would be worse than the remaining stroke participants.

This study enabled us to follow a scientific and valid process at both a theoretical and methodological level for adapting a diagnostic tool. Our findings revealed high reliability for the complete BIT (*α* = .90), as well as for the conventional (*α* = .93) and behavioral subtests (*α* = .75). Also, internal consistency was considered good to excellent. These findings contrast with those reported for the Turkish adaptation of the BIT [20], for which the conventional test was less valid (*α* = .77); however, the validity of the behavioral section was greater (*α* = .91), compared to our findings.

In addition, in our study, concurrent validity was observed between conventional and behavioral sections (*r* = −.80). The concurrent validity was less than that of the original BIT [21].

In the comparison between stroke patients with, and without, USN, our study showed significant differences between both groups in all the items on the BIT. According to the results obtained, there are statistically significant differences between subgroups in all the BIT-E variables, therefore enabling the conclusion that the BIT-E detects problems of neglect and attention problems between patients with and without unilateral spatial neglect. An Israeli version has also been adapted, reporting similar results in the behavioral test except for article reading and telling and setting the time, where no differences were found [22].

The translation procedure employed in this study is commonly used. The translation, cultural adaptation, and validation processes were based on the recommendations by Beaton et al. [19]. During the adaptation process, the translation of the tests that required words in Spanish did not give rise to discussion, given that the concepts were simple and easy to translate. The majority of the discussion focused on certain photographs and tests including Spanish food (Figure 1(a)), coins (*coin sorting *test), and objects (*card sorting* test). Certain simple nominated elements were added to Figures 1(b) and 1(c). In the prefinal version of the questionnaire used with a sample of stroke patients, the pilot study showed an optimal degree of understanding.

The BIT has also been translated and validated for other populations. The adaptation of the tool to the population of Turkey was carried out by applying the Rasch model. The reported findings showed a lack of reliability and validity of the internal construct, given that although, in certain aspects, the results were considered adequate, there were problems relating to certain tests that clearly failed to achieve the expectations of the model [20]. In contrast, the results of the Israeli adaptation of the test [22] support the predictive and construct validity in the majority of behavioral and functional BITs for measuring unilateral neglect. The number of participants in this study was smaller than those participating in the Turkish BIT adaptation (*n* = 118), although higher than the Israeli version (*n* = 40). The time since stroke onset varied considerably in our study compared to the Israeli study (mean: 11 months) and the Turkish study (median: 38 days). Our sample presents a greater chronicity in comparison with the remaining BIT adaptation studies and possibly the severity of the participants' neglect in the present study was greater.

### 4.1. Study Limitations

This study presents important limitations. Firstly, the sample comes from the same Spanish region (Community of Madrid). Furthermore, the number of women was higher than men, and the level of education of women was also higher; therefore, there could be a selection bias. Also, the psychometric properties of the scale were not analyzed in depth; thus data for test-retest reliability, construct validity, and interrater reliability is not presented at this time.

## 5. Conclusions

The BIT is a tool used to evaluate unilateral spatial neglect which provides information on everyday problems faced by patients who have suffered from a stroke. We translated the BIT into Spanish, elaborating a Spanish version conceptually equivalent to the English version which was understood by our patients with unilateral spatial neglect, thanks to the academic translation methods employed. The availability of an adapted and validated Spanish version of the tool (BIT-E) provides professionals with a reliable instrument that contributes to the effective diagnosis, planning, and design of individualized treatment interventions that will no doubt improve the functionality and quality of life of patients with unilateral spatial neglect.

## Figures and Tables

**Table 1:** Sociodemographic data. Complete sample.

	Left stroke	Right stroke	Total	Healthy controls	*p* value
*n*	17	58	75	18	
Age	53.29 ± 10.31	58.52 ± 12.58	55.77 ± 13.40	47.28 ± 15.25	0.011^*∗*^
Age range	39–79	32–91	23–91	26–74	
Sex					0.193
Male	5 (29.4%)	38 (65.5%)	43 (57.3%)	7 (38.9%)	
Female	12 (70.6%)	20 (34.5%)	32 (42.7%)	11 (61.1%)	
Education level					0.842
Basic	1 (5.9%)	8 (13.8%)	9 (12%)	2 (11.1%)	
Primary	8 (47.1%)	22 (37.9%)	30 (40%)	6 (33.3)	
Secondary	8 (47.1%)	28 (48.3%)	36 (48%)	10 (55.6%)	
With unilateral spatial neglect	4 (23.5%)	37 (63.8%)	41 (54.7%)	—	
Without unilateral spatial neglect	13 (76.5%)	21 (36.2%)	34 (45.3%)	—	

*Note.* Values are expressed as mean ± SD and frequency is expressed as a percentage (%). ^*∗*^Significant with *p* < 0.05. Differences between stroke patients and healthy controls.

**Table 2:** Data on stroke patients with and without unilateral spatial neglect.

	With USN	Without USN	*p* value
*n*	41	34	
Age	60.61 ± 11.76	53.38 ± 11.79	0.004^*∗*^
Age range	33–91	32–81	
Sex			0.035
Male	28 (68.3%)	15 (44.1%)	
Female	13 (31.7%)	19 (55.9%)	
Diagnosis of stroke	41 (93.2%)	34 (87.2%)	
Damaged side			0.003^*∗*^
Right	37 (90.2%)	21 (61.8%)	
Left	4 (9.8%)	13 (38.2%)	
Stroke onset (years)	3.20 ± 3.77	4.32 ± 4.70	0.124
Range of evolution time	1–14	0–19	

*Note.* Values are expressed as mean ± SD and frequency is expressed as a percentage (%). ^*∗*^Significant with *p* < 0.05. Differences between patients with Unilateral spatial neglect and without Unilateral spatial neglect.

**Table 3:** Results obtained by healthy control participants in conventional and behavioral tests (*n* = 18).

Subtest	Maximum score	Cut-off point (acceptable range)	Median (q1–q3)	Min–max	Cut-off score
*Conventional subtest*					
Line cancellation	36	34 (35-36)	36 (36-36)	23–36	34
Letter cancellation	40	32 (33–40)	40 (40-40)	36–40	32
Star cancellation	54	51 (52–54)	54 (54-54)	4–54	51
Copying figures and shapes	4	3 (4)	4 (4-4)	4–8	3
Line bisection	9	7 (8-9)	9 (8-9)	3–9	7
Drawing	3	2 (3)	3 (3-3)	3–9	2

Total Conventional subtest	146	129	146 (144.75–146)	132–146	129

*Behavioral subtest*					
Picture scanning	9	5 (6–9)	9 (8-9)	7–9	5
Phone dialing	9	7 (8-9)	9 (9-9)	9-9	7
Menu reading	9	8 (9)	9 (9-9)	9-9	8
Article reading	9	8 (9)	9 (9-9)	9-9	8
Telling and setting the time	9	8 (9)	9 (9-9)	9-9	8
Coin sorting	9	8 (9)	9 (9-9)	9-9	8
Address and sentence copying	9	7 (8-9)	9 (9-9)	9-9	8
Map navigation	9	8 (9)	9 (9-9)	9-9	7
Card sorting	9	8 (9)	9 (9-9)	9-9	8

Total behavioral subtest	81	67	81 (80-81)	79–81	67

*Note.* Values expressed as median (q1–q3).

**Table 4:** Descriptive data on scales and reliability (*n* = 75).

Scale	Median (q1–q3)	Min–max	*α*
Conventional Test	136 (115–142)	61–146	0.93
Behavioral Test	70 (50–76)	3–81	0.75

*Note.* Values expressed as median (q1–q3).

**Table 5:** Comparison of groups of patients with and without USN (*n* = 75).

	With USN	Without USN	*p* value
	Median (q1–q3)	Median (q1–q3)	
*Conventional test*	115 (93.5–132)	141 (138–144)	<0.001^*∗*^
Line cancellation	33 (24–36)	36 (36-36)	<0.001^*∗*^
Letter cancellation	30 (25–37.5)	38 (36.75–40)	<0.001^*∗*^
Star cancellation	44 (34–51)	53.5 (52–54)	<0.001^*∗*^
Copying figures and shapes	3 (0.5–4)	4 (3-4)	<0.001^*∗*^
Line bisection	5 (4–7.5)	8 (7–9)	<0.001^*∗*^
Drawing	2 (0–3)	3 (3-3)	<0.001^*∗*^
*Behavioral test*	50 (25.5–65.5)	75.5 (72.75–79)	<0.001^*∗*^
Picture scanning	3 (0–6)	7 (6–8)	<0.001^*∗*^
Phone dialing	8 (5.5–9)	9 (9-9)	<0.001^*∗*^
Menu reading	7 (1–9)	9 (9-9)	<0.001^*∗*^
Article reading	5 (0–9)	9 (9-9)	<0.001^*∗*^
Telling and setting the time	8 (6–9)	9 (8-9)	<0.001^*∗*^
Coin sorting	3 (1–7)	7 (7–9)	<0.001^*∗*^
Address and sentence copying	6 (0–9)	9 (9-9)	<0.001^*∗*^
Map navigation	7.5 (4–9)	9 (9-9)	<0.001^*∗*^
Card sorting	3 (1–6)	9 (9-9)	<0.001^*∗*^

*Note.* Values expressed as median (q1–q3). ^*∗*^Significant with *p* < 0.05. Differences between patients with unilateral spatial neglect and without unilateral spatial neglect.

**Table 6:** Comparison of groups according right or left damaged side (*n* = 75).

	Right stroke	Left stroke	*p* value
	Median (q1–q3)	Median (q1–q3)	
*Conventional test*	130.50 (102.25–140)	140 (136–144.5)	0.007^**∗**^
Line cancellation	36 (31–36)	36 (36-36)	0.028^**∗**^
Letter cancellation	35.50 (26–38)	38 (36–39)	0.033^**∗**^
Star cancellation	50 (38.75–53)	54 (52.5–54)	0.002^**∗**^
Copying figures and shapes	3 (2–4)	4 (3-4)	0.001^**∗**^
Line bisection	6.5 (4–8)	9 (7.5–9)	0.009^**∗**^
Drawing	2 (1–3)	3 (3-3)	0.002^**∗**^
*Behavioral test*	66.5 (42.75–74.25)	75 (67.5–78.5)	0.010^**∗**^
Picture scanning	5 (1–7)	8 (6.5–8.5)	<0.001^**∗**^
Phone dialing	9 (7–9)	9 (9-9)	0.009^**∗**^
Menu reading	8 (3–9)	9 (9-9)	0.006^**∗**^
Article reading	9 (3–9)	9 (7–9)	0.156
Telling and setting the time	8 (7–9)	9 (9-9)	0.005^**∗**^
Coin sorting	6 (3–7)	7 (7–9)	0.012^**∗**^
Address and sentence copying	9 (2–9)	9 (9-9)	0.018^**∗**^
Map navigation	9 (6.75–9)	9 (9-9)	0.009^**∗**^
Card sorting	6 (3–9)	9 (4.5–9)	0.117

*Note.* Values expressed as median (q1–q3). ^*∗*^Significant with *p* < 0.05. Differences between right and left stroke patients.
